# Anxiety and Depression in Newly Diagnosed Epilepsy: A Matter of Psychological History?

**DOI:** 10.3389/fneur.2021.744377

**Published:** 2021-10-05

**Authors:** Natacha Forthoffer, Alexis Tarrada, Hélène Brissart, Louis Maillard, Coraline Hingray

**Affiliations:** ^1^Laboratoire de Neurosciences Cognitives et Adaptatives (LNCA), Centre National de la Recherche Scientifique (CNRS), Université de Strasbourg, Strasbourg, France; ^2^Département de Neurologie, Centre Hospitalier Universitaire de Nancy, Nancy, France; ^3^Unité de Formation et de Recherche (UFR) Médecine Paris Centre, Université de Paris, Paris, France; ^4^Centre de Recherche en Automatique de Nancy (CRAN), Centre National de la Recherche Scientifique (CNRS), Université de Lorraine, Nancy, France

**Keywords:** epilepsy, newly diagnosed, anxiety, depression, new-onset

## Abstract

**Purpose:** Anxiety and depression are highly prevalent in patients with epilepsy (PWE), and these symptoms can even precede the onset of the pathology. We aimed to define the prevalence of anxiety and depressive symptoms at the time of the epilepsy diagnosis and the factors related to their presence in newly diagnosed adult patients.

**Methods:** One hundred and twelve newly diagnosed patients were assessed, usually in the week after diagnosis. Patients were untreated at this time. We used the Neurological Disorders Depression Inventory for Epilepsy (NDDI-E, cut-off ≥15) and the Generalized Anxiety Disorder 7-Item scale (GAD-7, cut-off >7). A semi-structured interview was conducted to collect sociodemographic and epilepsy data and patients' psychiatric history. We first compared patients with and without anxiety symptoms, then patients with and without depressive symptoms.

**Results:** According to the GAD-7 scale, the prevalence of anxiety symptoms at the time of diagnosis was 35%. Patients with anxiety symptoms had significantly more psychiatric history (26%, *p* = 0.001) and more history of psychological trauma (51%, *p* = 0.003) than patients with no anxiety symptoms. According to the NDDI-E scores, the prevalence of depressive symptoms at the time of the diagnosis was 11%. Patients with depressive symptoms had significantly more psychiatric history (43%, *p* < 0.001) and more history of psychological trauma (65%, *p* = 0.007) than patients with no depressive symptoms. No difference between groups was found for other sociodemographic variables (age and gender), epilepsy characteristics (number of seizures prior to diagnosis, time from first seizure to diagnosis, type of epilepsy, and localization in focal epilepsy), or neurological comorbidities.

**Conclusions:** Anxiety symptoms are common whereas depressive symptoms are less prevalent at the time of diagnosis. It appears essential to be aware of anxiety and depression in newly diagnosed epileptic patients. They should be screened and routinely monitored, especially those patients with a history of psychological trauma and/or psychiatric disorders. Longitudinal follow-up is required to identify whether these factors and anxiety and depression themselves have an impact on the future course of care.

## Introduction

Epilepsy is a chronic multifactorial neurological disease encompassing psychological factors, as described in the International League Against Epilepsy (ILAE) definition: “epilepsy is a disorder of the brain characterized by an enduring predisposition to generate epileptic seizures and by the neurobiological, cognitive, psychological, and social consequences of this condition” (1). The ILAE definition highlights the relevance of considering the psychological dimension of epilepsy.

Patients with epilepsy (PWE) also have a higher risk of developing psychiatric comorbidities, which can affect one-third of patients (2). Compared with controls, PWE are more likely to suffer from depression, with a relative risk ranging from 1.43 (3) to 2.7 (4). The estimated prevalence of depression is around 23.1% and comorbid anxiety affects 20.2% of PWE (5, 6). These studies looked at all types of epilepsy combined (drug-resistant and drug-sensitive epilepsy), whereas the risk of experiencing psychiatric comorbidities is four times higher in drug-resistant epilepsy (7, 8). Conversely, people with mood and anxiety disorders have a seven-fold increase in the risk of developing epilepsy (9–13).

These data highlight a bidirectional correlation between epilepsy and mood/anxiety disorder (14). Psychiatric disorders in epilepsy have specific characteristics that clinicians need to consider at the time of assessment (6). Psychiatric symptoms are classified according to their temporal relationship to the seizures: periictal (chronologically linked with a seizure) or interictal symptoms (no chronological link with a seizure) (15). It is noteworthy that psychiatric disorders are associated with impaired quality of life (QoL) and increased frequency of seizures (16, 17). Consideration and assessment of psychiatric comorbidities is therefore essential. In this study, we propose to do this as early as possible in the history of epilepsy.

Anxiety and depressive disorders have been extensively studied in drug-resistant epileptic patients who often have longstanding epilepsy and still have seizures. This is a specific population, and the results cannot be extended to all PWE. There are fewer studies in drug-sensitive or newly diagnosed patients (18–21). We know that anxiety and depression can even precede the onset of the epilepsy (22): in a population of 3,773 PWE and 14,025 matched controls, the PWE were found to be more likely to have depression, notably during the 3 years preceding the diagnosis of epilepsy. There thus appears to be a clear bidirectional link between psychiatric comorbidities and epilepsy. Screening for anxiety and depression should therefore be conducted at an early stage.

Studies report a prevalence of anxiety ranging from 19 to 42.6% in newly diagnosed epilepsy (NDE) (18–21). The prevalence of depression varies from 11 to 44.7% in this population (18–21). There is therefore a wide disparity in prevalence between studies, which is surely related to the populations studied [NDE, new-onset epilepsy (NOE), and/or first seizure], the different tools used [mostly the Hospital Anxiety and Depression Scale (HADS) but also the Neurological Disorders Depression Inventory for Epilepsy (NDDI-E), and the Generalized Anxiety Disorder-7 (GAD-7) scale], and the cut-off points chosen [e.g., 14 for GAD-7 in the study by Lane et al. (19)].

To our knowledge, the factors related to anxiety and depression at the time of epilepsy diagnosis have not been extensively studied (18, 21).

Anti-epileptic drugs (AEDs) are well-known to induce psychiatric disorders in PWE (23–25). Up to 15% of patients can be expected to have psychiatric symptoms of iatrogenic origin (26). Antiepileptic drugs induce several types of psychiatric disorders, including anxiety, depression, irritability, hallucinations, and delusions. These symptoms vary in duration and occur from a few hours to several weeks after the introduction of treatment (15). This is a major confounding factor that is difficult to control in PWE studies. The opportunity to focus on newly diagnosed patients who are not currently taking AEDs is therefore novel and valuable.

We aimed to define the prevalence of anxiety and depression symptoms and their related factors in a sample of adult patients with NDE who were not being treated with AED.

## Materials and Methods

### Study Design and Participants

This is an observational prospective monocentric study. Participants were recruited from Nancy Hospital Epilepsy Unit between June 2017 and March 2021. The study design was approved by the Regional Ethical Standards Committee on Human Experimentation (France, CPP no. 20.07.23.36832). Subjects were aged over 16 years and had NDE. Diagnosis and syndrome classification were based on clinical history and electroencephalogram findings according to the ILAE (27). Newly diagnosed epilepsy means that diagnosis is recent, but seizures may have gone unrecognized for over a year. We considered NDE to include NOE, which corresponds to the onset of seizures within the last year. Patients were excluded if they had experienced provoked seizures, had a history of drug/alcohol misuse, or had previously used AEDs. All patients gave their non-opposition for inclusion in this study.

### Data Collected

Patients were assessed at the time of diagnosis (during the week after diagnosis) and were untreated at this time.

#### Demographic Data

Demographic data were collected by means of an interview, in which we gathered information on age, gender, and level of education.

#### Epilepsy Data

We collected classification of seizure type (focal with lateralization, or generalized), number of seizures prior to diagnosis, time between the first seizure and diagnosis, and lesions on MRI.

#### Neurological Data

We collected data on patient MRIs and neurological comorbidities (stroke, traumatic brain injury, etc.).

#### Psychiatric Data

Psychiatric data were obtained with particular focus on psychiatric history and treatment. A semi-structured interview was conducted, with questions addressing patients' history of depression, anxiety disorders, suicide attempts, use of anti-depressants, medication for anxiety and/or psychotherapy, and psychiatric hospitalization. We considered that there was a mentioned psychiatric history if the patient refers any one of these issues.

We also actively screened for traumatic events (natural disasters, accident, deliberate violence, abuse, harassment, sudden death, etc.).

#### Validated Scales in Epilepsy

We used the NDDI-E (28) and the GAD-7 (29) to investigate the presence of depressive and anxiety symptoms, respectively. The NDDI-E and the GAD-7 are both validated screening tools for such symptoms in epilepsy (29). We used cut-off scores of 15 for the NDDI-E and 7 for the GAD-7, forming our study groups according to these scales. Participants with an NDDI-E score of over 15 and a GAD-7 score of over 7 were included in the depressive symptoms group and the anxiety symptoms group, respectively.

#### Other Psychometric Scales

In addition, we used the State-Trait Anxiety Inventory (STAI) to measure patients' state and trait anxiety. A score of 35 or less is considered very low, 36–45 low, 46–55 medium, 56–65 high, and above 65, very high. For depression, we also used the Beck Short Form Depression Inventory (BDI). A score of 0–4 indicates no depression, 4–7 indicates mild depression, 8–15 indicates moderate depression, and 16 and above indicates severe depression.

### Data Analyses

We compared demographic data, medical data, and psychiatric data according to the presence or absence of depressive or anxiety symptoms at the time of diagnosis. Sociodemographic and clinical data are displayed as mean, SD, and median (med) for numeric variables. For nominal variables, we used patient numbers or percentages. Differences in mean values were calculated using Student's *t*-test (after ensuring normality and equality of variance) or the Mann–Whitney *U*-test for independent samples. For nominal variables, we used the χ^2^-test or Fisher's exact test. *P*-value ≤ 0.05 was considered significant. Data were analyzed using Jamovi 1.6.15.

## Results

### Description of Cohort

One hundred and twelve patients were included. Seventy-six patients were enrolled prior to the worldwide COVID pandemic. As summarized in Table 1, 62 females and 50 males were included, with a mean age of 45.11 ± 21.38 years and a mean duration of education of 11.93 ± 2.47 years.

**Table 1:** Sociodemographic and medical data (*n* = 112).

Gender (*n*, %)	
Male	50 (45%)
Female	62 (55%)
Age [mean (SD)]	45.11 (21.38)
Duration of education [mean (SD)]	11.93 (2.47)
Time between first seizure and diagnosis in months [mean (SD)]	19.14 (44.68)
Number of seizures before diagnosis [mean (SD)]	42.33 (208.95)
New-onset epilepsy (*n*, %)	76 (68%)
Newly diagnosed epilepsy (*n*, %)	36 (32%)
Type of epilepsy (*n*, %)	
Focal	87 (80%)
Generalized	22 (20%)
Lateralization of epilepsy in focal epilepsy (*n*, %)	
Left	40 (59%)
Right	28 (41%)
Neurological comorbidities (*n*, %)	
Yes	40 (36%)
No	82 (64%)
Lesions on MRI (*n*, %)	
Yes	38 (40%)
No	59 (60%)
Psychiatric history (*n*, %)	
Yes	13 (12%)
No	97 (88%)
Psychological trauma mentioned (*n*, %)	
Yes	35 (33%)
No	72 (67%)
Anxiety symptoms (*n*, %)	
GAD-7 >7	39 (35%)
State STAI ≥46	16 (16.5%)
Trait STAI ≥46	28 (29%)
Depressive symptoms (*n*, %)	
NDDI-E ≥15	14 (12.5%)
Short-form BDI ≥10	11 (11%)

#### Epileptic Data

Eighty percent of patients had focal epilepsy (41% right-sided and 76% temporal) and 68% had NOE. The mean time between first seizure and diagnosis was 19.14 ± 44.68 months, but the median was 6. The mean number of seizures before diagnosis was 42.33 ± 208.95, but the median was 3. The majority of patients had only focal seizures (68%). Generalized seizures were less frequent and most patients had <4 (mean of 1) except for two patients who had, respectively, 10 and 52 absence-type seizures before diagnosis.

#### Medical Data

Forty percent of patients had a lesion found on MRI (atrophy, vascular damage, cavernoma, dysplasia, cyst, polymicrogyria, etc.) and 36% of patients had a neurological comorbidity (stroke, traumatic brain injury, etc.).

#### Psychiatric Data

Twelve percent of patients had psychiatric history: nine had depression (two with a suicide attempt), one had experienced burn-out, one had a generalized anxiety disorder, one had an eating disorder, and one had post-traumatic stress disorder. Four of these patients are currently being treated with psychopharmacological treatments. No other patients in this study are being treated with this medication.

Thirty-three percent of patients mentioned a history of psychological trauma. Most (14 patients) cited interpersonal violence (domestic violence, harassment, stabbing, child abuse, etc.), the sudden death of a relative, or a serious illness leading to death (13 patients). Seven patients also cited accidents (car, train) and one cited a natural disaster (fire).

The prevalence of anxiety symptoms at the time of diagnosis was 35% according to the GAD-7 scale and was quite similar for patients included before and after the start of the COVID pandemic (*p* = 0.913). According to the STAI scale, state anxiety had a prevalence of 16.5% at the time of diagnosis and trait anxiety, 29%.

The prevalence of depression symptoms at the time of diagnosis was 12.5% according to the NDDI-E and was quite similar for patients included before and after the start of the COVID pandemic (*p* = 0.375). At the time of diagnosis, 11% of patients had symptoms of depression according to the short-form BDI.

### Comparison of Groups According to Anxiety Symptoms

We compared patients with anxiety symptoms (defined by the GAD-7 score) (*n* = 39) and without (*n* = 73) such symptoms. The presence of psychiatric history (26 vs. 4%, *p* = 0.001) and at least one psychological trauma (51 vs. 23%, *p* = 0.003) was overrepresented in patients with anxiety symptoms (Table 2). We found no difference in other sociodemographic variables (age and gender) or in the characteristics of the epilepsy [number of seizures prior to diagnosis, time from first seizure to diagnosis, side (*p* = 0.462), and type of epilepsy].

**Table 2:** Sociodemographic and medical data according to the presence or absence of anxiety symptoms.

	**Patients with anxiety symptoms**	**Patients without anxiety symptoms**	***p***
	**(*n* = 39)**	**(*n* = 73)**	
Gender (*n*, %)			
Male	13 (33%)	37 (51%)	0.078^a^
Female	26 (67%)	36 (49%)	
Age [mean (SD)]	43.2 (19.71)	46.2 (22.28)	0.482^b^
(med)	41	46	
Duration of education [mean (SD)]	12 (2.54)	11.9 (2.44)	0.887^b^
(med)	12	11	
Time between first seizure and diagnosis in months [mean (SD)]	16.9 (23.59)	20.3 (52.53)	0.710^b^
(med)	6	6	
Number of seizures before diagnosis [mean (SD)]	97.6 (351.21)	13.9 (38.18)	0.903^c^
(med)	3	3	
New-onset epilepsy (*n*, %)	26 (67%)	50 (68.5%)	0.844^a^
Newly diagnosed epilepsy (*n*, %)	13 (33%)	23 (31.5%)	
Type of epilepsy (*n*, %)			
Focal	28 (78%)	59 (81%)	0.710^a^
Generalized	8 (22%)	14 (19%)	
Lateralization of epilepsy in focal epilepsy (*n*, %)			
Left	11 (48%)	29 (64%)	0.188^a^
Right	12 (52%)	16 (36%)	
Neurological comorbidities (*n*, %)			
Yes	14 (36%)	22 (30%)	0.534^a^
No	25 (64%)	51 (70%)	
Lesions on MRI (*n*, %)			
Yes	13 (38%)	25 (40%)	0.889^a^
No	21 (62%)	38 (60%)	
Psychiatric history (*n*, %)			
Yes	10 (26%)	3 (4%)	0.001^*^^d^
No	28 (74%)	69 (96%)	
Psychological trauma mentioned (*n*, %)			
Yes	19 (51%)	16 (23%)	0.003^*^^a^
No	18 (49%)	54 (77%)	

* *p < 0.05*;

a *Chi-square test*;

b *Student's t-test*;

c *Mann-Whitney U-test*;

d *Fisher's exact test*.

### Comparison of Groups According to Depressive Symptoms

We compared patients with depressive symptoms (as defined by their NDDI-E scores) (*n* = 14) and without (*n* = 98) such symptoms. The presence of a psychiatric history (43 vs. 7%, *p* < 0.001) and psychological trauma (64 vs. 28%, *p* = 0.007) was over-represented in patients with depressive symptoms (Table 3). We found no difference in other sociodemographic variables (age and gender) or in the characteristics of the epilepsy [number of seizures prior to diagnosis, time from first seizure to diagnosis, side (*p* = 0.297), and type of epilepsy].

**Table 3:** Sociodemographic and medical data according to the presence or absence of depressive symptoms.

	**Patients with depressive symptoms**	**Patients without depressive symptoms**	***p***
	**(*n* = 14)**	**(*n* = 98)**	
Gender (*n*, %)			
Male	4 (29%)	46 (47%)	0.256^d^
Female	10 (71%)	52 (53%)	
Age [mean (SD)]	39.9 (16.77)	45.9 (21.93)	0.328^b^
(med)	39	45.9	
Duration of education [mean (SD)]	12.6 (2.5)	11.8 (2.46)	0.249^b^
(med)	12	11	
Time between first seizure and diagnosis in months [mean (SD)]	23.4 (23.48)	18.5 (47.01)	0.708^b^
(med)	11.5	6	
Number of seizures before diagnosis [mean (SD)]	172.7 (551.51)	24.1 (86.13)	0.375^c^
(med)	3	3	
New-onset epilepsy (*n*, %)	7 (50%)	69 (70%)	0.126^a^
Newly diagnosed epilepsy (*n*, %)	7 (50%)	29 (30%)	
Type of epilepsy (*n*, %)			
Focal	11 (79%)	76 (80%)	0.901^d^
Generalized	3 (21%)	19 (20%)	
Lateralization of epilepsy in focal epilepsy (*n*, %)			
Left	4 (40%)	36 (62%)	0.297^d^
Right	6 (60%)	22 (38%)	
Neurological comorbidities (*n*, %)			
Yes	6 (43%)	30 (31%)	0.359^a^
No	8 (57%)	68 (69%)	
Lesions on MRI (*n*, %)			
Yes	7 (54%)	31 (37%)	0.244^a^
No	6 (46%)	53 (63%)	
Psychiatric history (*n*, %)			
Yes	6 (43%)	7 (7%)	<0.001^*^^a^
No	8 (57%)	89 (93%)	
Psychological trauma mentioned (*n*, %)			
Yes	9 (64%)	26 (28%)	0.007^*^^a^
No	5 (36%)	67 (72%)	

* *p < 0.05*;

a *Chi-square test*;

b *Student's t-test*;

c *Mann-Whitney U-test*;

d *Fisher's exact test*.

#### Supplementary Data

Supplementary data provides results for patients with no neurological comorbidities (82 patients). This research will be extended to the study of cognitive aspects, for which the separation is important. There are no significant differences for this group between patients with or without anxiety symptoms. However, the presence of a psychiatric history (*p* = 0.027) and psychological trauma (*p* = 0.035) was still over-represented in patients with depressive symptoms.

## Discussion

The main findings of this study show that patients who have experienced psychological trauma or who have a psychiatric history are more likely to present anxiety and depressive symptoms at the time of diagnosis. It is important to emphasize that patients were not being treated with AED at the time of the assessment, to exclude AED-induced depression or anxiety.

### Prevalence of Depressive and Anxiety Symptoms

In this study, we assess the prevalence of anxiety and depressive symptoms in NDE patients. The prevalence of such symptoms found in our sample at the time of diagnosis is consistent with previous studies in the same population (18–21). It is noteworthy that the percentage is close to that recorded in a study of patients with drug-resistant focal epilepsy (6). Jansen et al. found that 31% of patients had psychiatric disorders even before the onset of the epilepsy. It is therefore necessary to follow up our patients to determine whether patients with these mood disorders are more likely to become drug resistant.

Interestingly, there is a significant difference between the prevalence of anxiety and depressive symptoms in our sample, whereas in most other studies they are quite similar (18, 19, 21). In these studies, anxiety and depression were mainly assessed using the HADS (18, 21). Notably in the study by Lee et al. (18), anxiety and depression were assessed using the HADS and their prevalence was similar. With other tools, Lane et al. found a higher prevalence of depression symptoms (33%) with anxiety symptoms occurring less frequently (23%) (19). This study was based on patients who had experienced potentially epileptic events but who had not received a diagnosis of epilepsy. Although they used the GAD-7 and the NDDI-E, the cut-off used for the GAD-7 was 14, which may have influenced the scores recorded (19). Moreover, in this study, 30% of the patients had regularly taken psychoactive substances.

Our results therefore confirm the presence of anxiety and/or depressive symptoms at the onset of epilepsy, or even beforehand. The issue of precise time frame is really complex to address because patients have to retrieve their information retrospectively. Longitudinal follow-up will enlighten us on this issue.

Qualitatively, several close relatives of the patients in this sample were able to report changes in their partner or child (irritability, changes of mood such as melancholia) in the previous year, even though no particular event had occurred. The patients themselves were sometimes very confused by these changes, which they could not explain. They did not report a link with the seizure(s) they experienced, especially since such changes may have occurred before the seizure(s). As an example, one patient said that he had had regular mood swings without understanding the underlying reason within the last year, even before the first seizure. There was no change in his everyday life. Mood swings in PWE, especially temporal lobe epilepsy, is a known disorder described by Blumer, so-called interictal dysphoric disorder (30).

Gender did not emerge significantly, although there is a tendency for patients with anxiety to be mainly female. This gender difference has been reported in the general population in anxiety and depressive disorders (31, 32) but not in PWE (33). Our results are therefore in line with those obtained previously in other studies. Unexpectedly, our result as regards gender did not achieve significance for depressive symptoms. This is inconsistent with studies on this topic, which suggest that women report more depressive symptoms than men in PWE (33). This result can be explained by the small number of patients with depressive symptoms (*n* = 14), which makes the statistical analyses less powerful. Further investigation with more patients is required. This finding could also imply that there is no gender-related difference at an initial stage of the disease and that this difference occurs as the disease progresses. This will be explored in the longitudinal follow-up of these patients.

### Specificity of Patients With a History of Psychological Trauma and Psychiatric Disorders

In our sample, 33% of patients reported psychological trauma, which appeared to be related to the occurrence of anxiety and/or depressive symptoms at the time of diagnosis. The link between psychological trauma and psychiatric disorders is well-established (34). We also know that psychological trauma may influence epileptogenesis (35). For example, patients with post-traumatic stress disorder are 3.7 times more likely to develop epilepsy than age- and gender-matched controls (36). Moreover, psychological trauma can influence the course of epilepsy and potentially its severity. For example, the rate of psychological trauma is higher in our previous study with focal drug-resistant epileptic patients (42.5%) (6). Seizure frequency is greater in children with epilepsy living in war zones than in those living in peaceful areas (37). Hence, screening for history of psychological trauma can be particularly relevant in patients at the onset of their epilepsy. Patients with a history of psychotrauma may also be at higher risk for depressive and/or anxious symptoms when a diagnosis of epilepsy is confirmed. Such an announcement can be perceived as a very stressful experience.

We also found that previous psychiatric comorbidity was implicated in the occurrence of anxiety and/or depressive symptoms in NDE. We assume that probably such symptoms may be a consequence of previous psychiatric disorders. We can surmise that these symptoms reflect the patient's psychiatric history (residual symptoms, active symptomatology, vulnerability induced by previous disorders). However, in patients with no mentioned psychiatric history but with anxiety and depressive symptoms, we cannot exclude that these symptoms are the potential expression of an underlying undiagnosed psychiatric disorder. The time frame and the recording of the free interval could not be obtained easily due to the memory bias of patients.

### Evaluation and Information

Considering the high prevalence of anxiety and depressive symptoms compared with the normal population, routine screening as recommended by guidelines is essential (2, 38). There are a number of validated screening tools such as the GAD-7 and the NDDI-E (28, 29) that are suitable for PWE. These scales are concise and easy to use, allowing for their adoption by all clinicians. They act as a mediator between the patient and the clinician, providing the patient with the opportunity to mention things that they would not have expressed spontaneously. It is equally important to provide patients and their relatives with information and to provide psycho-education right from the first consultation. This can help to eliminate any stigmas that may be developing and have an impact on these patients' psychiatric comorbidities (18). Furthermore, the information should be given routinely as psychiatric comorbidities in epilepsy patients influence QoL and seizure outcome (16, 17).

It is therefore crucial to assess this aspect, to follow up, in particular, those patients with psychiatric comorbidities and who have experienced psychological trauma, and to advise them of the considerable adverse impact that such comorbidities may have on the outcome of their epilepsy.

During the assessment, some patients mentioned having a fear of being judged because they have epilepsy. They also mention the fear of the consequences that this could have on their work, especially since they can no longer drive. The emergence of these fears at the time of diagnosis must be explored and followed up.

There is a clear issue of early identification and prompt treatment of psychiatric and psychological aspects (2).

## Limitations

Our study has several limitations. The number of patients is small in some cases due to division into several groups for statistical analysis. A larger sample size will also lead to more powerful statistical analysis.

Moreover, the assessment was most often conducted within a week of diagnosis. Anxiety related to the diagnosis may still be very marked and may increase the prevalence of anxiety in our sample. We are aware that this epileptic pathology can have a negative connotation and carry stigma. This can lead to negative social relationships. In addition, epilepsy often implies a decrease of employability, or even to a driving prohibition. These things can cause major anxiety at the time of diagnosis. One advantage of this timing is that these patients were not yet on antiepileptic drugs when they were included. It also provided an opportunity for remote psychoeducation and allowed patients to ask any questions they had about epilepsy.

We did not have the exact duration between the start of a thymic disorder and the epilepsy because most of the patients cannot find the time period themselves.

In our study, we have not assessed particularly ictal anxiety (such as fear and agitation that have occasionally been reported in PWE as the only manifestation of focal seizures) (39). We are well-aware that it can be observed in focal epilepsies, especially in temporal epilepsy, but we decided to focus on interictal anxiety assessed by the GAD-7.

About a third of the patients were enrolled after the onset of the COVID pandemic, but this does not seem to have had an impact on the prevalence of depressive and anxiety symptoms.

## Perspectives

Findings of psychiatric comorbidities at the time of diagnosis and even beforehand need to be recognized by neurologists when the epilepsy diagnosis is confirmed, as well as by psychiatrists who need to be aware that epilepsy may be preceded by symptoms of depression and anxiety. We focused on the prevalence of depressive and anxiety symptoms at the time of diagnosis, but it will be necessary to follow these patients over time and ascertain which factors are associated with their presence and especially their persistence over time. There are few long-term longitudinal studies and more are required to provide answers to these questions. These studies show that the prevalence of depression and anxiety decreases 1 year after diagnosis. Sociodemographic factors do not appear to be the best predictors of the persistence of these comorbidities; this role is fulfilled by psychiatric history and seizure frequency, however (18, 20, 21). Psychiatric history was mentioned in only one study in NDE, and it encourages us to question patients more thoroughly about psychological trauma and psychiatric history (20). We assume that patients who already have a psychiatric history are more mentally vulnerable. Indeed, once the patient is treated and recovers, he or she often achieves a healthy balance that allows him or her to return to a normal life. Epilepsy disrupts the state of mind they had previously achieved, making them more sensitive, and increasing the presence of depressive and anxiety symptoms. Longitudinal follow-up of these patients will help us to identify whether experiencing a traumatic event is a contributing factor to the development of drug-resistant epilepsy.

The decrease in the prevalence of anxiety disorders at 1 year after diagnosis described in the literature may highlight a normal process of anxiety in response to the diagnosis (18, 21). These studies used HADS to assess depression and anxiety. It would be interesting to analyze in more detail the specificity of our screening tools for the NDE population in particular.

This evidence of the possible presence of anxiety/mood disorders in NDE should be recorded by all clinicians working with this population and especially by neuropsychologists. Indeed, as cognitive assessment of NDE patients is increasingly frequent, these psychiatric aspects need to be considered, especially as we know that they can have an impact on cognitive performance [see Forthoffer et al. for a brief review (40)].

## Conclusion

Anxiety symptoms are common whereas depressive symptoms are less prevalent at the time of diagnosis. Patients with psychological trauma or with a psychiatric history are more likely to present anxiety and depressive symptoms at the time of diagnosis. The number of seizures prior to diagnosis and the type of epilepsy appeared to be unrelated to depressive and anxiety symptoms. It seems essential to be aware of anxiety and depression in NDE patients. They should be screened and need to be routinely monitored, especially those patients who have experienced psychological trauma and/or who have a psychiatric history. Longitudinal follow-up is required to identify whether these factors and anxiety and depression themselves have an impact on the future course of care.

## Data Availability Statement

The original contributions presented in the study are included in the article/Supplementary Material, further inquiries can be directed to the corresponding author/s.

## Ethics Statement

The studies involving human participants were reviewed and approved by Comité de Protection des Personnes Sud-Est IV no. 20.07.23.36832. Written informed consent to participate in this study was provided by the participants' legal guardian/next of kin.

## Author Contributions

NF: helped in initiating and designing the study, drafting the paper, and deciding on the analytical strategy. AT: provided a critical review of the draft. HB: provided a critical review of the draft. LM: provided a critical review of the draft. CH: assisted with data management and drafting the paper. All authors contributed to the article and approved the submitted version.

## Funding

The authors declare that this study received funding from UCB Pharma. The funder was not involved in the study design, collection, analysis, interpretation of data, the writing of this article or the decision to submit it for publication.

## Conflict of Interest

The authors declare that the research was conducted in the absence of any commercial or financial relationships that could be construed as a potential conflict of interest.

## Publisher's Note

All claims expressed in this article are solely those of the authors and do not necessarily represent those of their affiliated organizations, or those of the publisher, the editors and the reviewers. Any product that may be evaluated in this article, or claim that may be made by its manufacturer, is not guaranteed or endorsed by the publisher.
