# Supplementary material for: Anxiety and Depression in Newly Diagnosed Epilepsy: A Matter of Psychological History?
Source: Front Neurol. 2021 Oct 5;12:744377. doi: 10.3389/fneur.2021.744377 (PMC8525707; doi:10.3389/fneur.2021.744377)
Supplement: Supplementary file 1 [file Table_1.docx]

| Table S1. Sociodemographic and medical data according to the presence or absence of anxiety symptoms in patients with no neurological comorbidities. | | | |
| --- | --- | --- | --- |
|  | **Patients with anxiety symptoms**  **(n=28)** | **Patients without anxiety symptoms**  **(n=54)** | **p** |
| Gender (n, %)  Male  Female | 12 (43%)  16 (57%) | 23 (43%)  31 (57%) | **0.982^a^** |
| Age (mean (SD))  (med) | 43.3 (21.10)  41.5 | 38.9 (19.06)  32.5 | **0.337^b^** |
| Duration of education (mean (SD))  (med) | 11.7 (2.33)  11 | 12.5 (2.30)  12 | **0.140^b^** |
| Time between first seizure and diagnosis in months (mean (SD))  (med) | 18 (24.73)  6 | 21.4 (50.74)  7 | **0.743^b^** |
| Number of seizures before diagnosis (mean (SD))  (med) | 21.5 (32.29)  5 | 27.2 (106.64)  2 | **0.788^b^** |
| New-onset epilepsy (n, %)  Newly diagnosed epilepsy (n, %) | 18 (64%)  10 (36%) | 35 (65%)  19 (35%) | **0.962^a^** |
| Type of epilepsy (n, %)  Focal  Generalized | 22 (85%)  4 (15%) | 38 (72%)  15 (28%) | **0.269^d^** |
| Lateralization of epilepsy in focal epilepsy (n, %)  Left  Right | 11 (58%)  8 (42%) | 16 (55%)  13 (45%) | **0.853^a^** |
| Lesions on MRI (n, %)  Yes  No | 9 (35%)  17 (65%) | 10 (22%)  35 (78%) | **0.935^a^** |
| Psychiatric history (n, %)  Yes  No | 4 (15%)  22 (85%) | 8 (15%)  46 (85%) | **1.000^d^** |
| Psychological trauma mentioned (n, %)  Yes  No | 8 (33%)  16 (67%) | 15 (28%)  38 (72%) | **0.655^a^** |

*=p<0.05; a=Chi-square test; b=Student’s t test; c=Mann-Whitney U test; d=Fisher’s exact test
