# Supplementary material for: Anxiety and Depression in Newly Diagnosed Epilepsy: A Matter of Psychological History?
Source: Front Neurol. 2021 Oct 5;12:744377. doi: 10.3389/fneur.2021.744377 (PMC8525707; doi:10.3389/fneur.2021.744377)
Supplement: Supplementary file 2 [file Table_2.docx]

| Table S2. Sociodemographic and medical data according to the presence or absence of depressive symptoms in patients with no neurological comorbidities. | | | |
| --- | --- | --- | --- |
|  | **Patients with depressive symptoms**  **(n=10)** | **Patients without depressive**  **Symptoms**  **(n=72)** | **p** |
| Gender (n, %)  Male  Female | 4 (40%)  6 (60%) | 35 (49%)  37 (51%) | **0.741^d^** |
| Age (mean (SD))  (med) | 37.8 (18.19)  32.50 | 40.7 (20.33)  37.50 | **0.672^b^** |
| Duration of education (mean (SD))  (med) | 12.7 (2.41)  12.50 | 12 (2.27)  12 | **0.348^b^** |
| Time between first seizure and diagnosis in months (mean (SD))  (med) | 18.4 (23.62)  8 | 18.3 (42.34)  6 | **0.996^b^** |
| Number of seizures before diagnosis (mean (SD))  (med) | 26.0 (62.76)  3 | 24.0 (91.14)  3 | **0.950^b^** |
| New-onset epilepsy (n, %)  Newly diagnosed epilepsy (n, %) | 6 (60%)  4 (40%) | 49 (68%)  23 (32%) | **0.723^d^** |
| Type of epilepsy (n, %)  Focal  Generalized | 7 (70%)  3 (30%) | 52 (75%)  17 (25%) | **0.708^d^** |
| Lateralization of epilepsy in focal epilepsy (n, %)  Left  Right | 1 (17%)  5 (83%) | 24 (60%)  16 (40%) | **0.079^d^** |
| Lesions on MRI (n, %)  Yes  No | 3 (33%)  6 (67%) | 13 (21%)  48 (79%) | **0.417^d^** |
| Psychiatric history (n, %)  Yes  No | 4 (40%)  6 (60%) | 7 (10%)  63 (90%) | **0.027^*,d^** |
| Psychological trauma mentioned (n, %)  Yes  No | 6 (60%)  4 (40%) | 18 (27%)  49 (73%) | **0.035^*,a^** |

*=p<0.05; a=Chi-square test; b=Student’s t test; c=Mann-Whitney U test; d=Fisher’s exact test
